# Normalized sensitivity of multi-dimensional body composition biomarkers for risk change prediction

**DOI:** 10.1038/s41598-022-16142-1

**Published:** 2022-07-20

**Authors:** A. Criminisi, N. Sorek, S. B. Heymsfield

**Affiliations:** 1 Amazon, Inc., Cambridge, UK; 2 Amazon, Inc., Tel Aviv, Israel; 3 Amazon, Inc., 2121 7th Avenue, Seattle, WA 98121 USA; 4 Pennington Biomedical Research Center, Louisiana State University System, Baton Rouge, USA

**Keywords:** Biomarkers, Risk factors

## Abstract

The limitations of BMI as a measure of adiposity and health risks have prompted the introduction of many alternative biomarkers. However, ranking diverse biomarkers from best to worse remains challenging. This study aimed to address this issue by introducing three new approaches: (1) a calculus-derived, normalized sensitivity score (NORSE) is used to compare the predictive power of diverse adiposity biomarkers; (2) multiple biomarkers are combined into multi-dimensional models, for increased sensitivity and risk discrimination; and (3) new visualizations are introduced that convey complex statistical trends in a compact and intuitive manner. Our approach was evaluated on 23 popular biomarkers and 6 common medical conditions using a large database (National Health and Nutrition Survey, NHANES, N ~ 100,000). Our analysis established novel findings: (1) regional composition biomarkers were more predictive of risk than global ones; (2) fat-derived biomarkers had stronger predictive power than weight-related ones; (3) waist and hip are always elements of the strongest risk predictors; (4) our new, multi-dimensional biomarker models yield higher sensitivity, personalization, and separation of the negative effects of fat from the positive effects of lean mass. Our approach provides a new way to evaluate adiposity biomarkers, brings forth new important clinical insights and sets a path for future biomarker research.

## Introduction

Body composition is associated with cardiorespiratory fitness and longitudinal health outcomes^1,2^. Excess adiposity impairs functional performance, is a major risk factor for developing chronic diseases, and is often accompanied by poor self-esteem^3–7^. The increased risk of chronic diseases that accompany excessive fat accumulation is the leading cause of death globally and contributes to an estimated $210 billion in medical costs in the US annually^8,9^.

In clinical practice, health risk levels are defined using body mass index (BMI), where adults with BMI ≥ 25 and ≥ 30 kg/m^2^ are classified as overweight and obese, respectively^10–12^. However, BMI cannot discern the fat component of body mass from lean tissues, which often leads to risk level misclassification^13,14^.

Alternative biomarkers are being designed that focus on global or regional body fat and lean mass, rather than weight. Some of these biomarkers use raw measurements such as waist circumference (WC)^15^. Others combine measurements together into ratios or more complex formulae, as for example Percentage Body Fat (PBF)^16^, Waist-to-Hip Ratio (WHR)^3^, fat-free mass index (FFMI)^17^, A Body Shape Index (ABSI)^18^ and Relative Fat Mass (RFM)^19^. However, the important question remains which biomarker or *combination* of biomarkers is best at predicting health risks, and for which condition.

This paper presents a new, simple and effective technique to assess existing biomarkers and their combinations, in terms of their risk predictive power.

Popular techniques to assess biomarkers use a simple disease classifier/detector obtained by thresholding a biomarker value, and measurements extracted from the associated confusion matrix (aka contingency table)^20^. Specificity, sensitivity and area under the ROC curve (also called c-statistics) are some of the most common such measurements^21–25^. Those approaches try to design or assess biomarkers that work well for detecting disease. In such prior work, the focus is on answering the question “Does my patient have condition C or not?”.

In contrast, our study aimed to answer a different question: “What body composition biomarker should I change, and by how much to achieve the largest reduction in my health risks?”. This question is most naturally answered by using differential calculus^26^. We introduce the Normalized Sensitivity score (NORSE), a new measure of risk *change* that is based on the established mathematical tool of sensitivity analysis^27^. Note that here the term “sensitivity” is intended as the rate of change of a dependent variable with respect to an independent one^28,29^ and is different from sensitivity as the True Positive Rate of a classifier^21^. Because of its focus on risk changes, the NORSE tool may be used to help people to take up healthier lifestyles and behaviors.

Unlike existing detection-based approaches, NORSE is designed to measure the *rate of change* of a health risk (e.g., prevalence of hypertension) with respect to changes in an input biomarker (e.g., body fat mass). We do not propose new biomarkers, rather a new way of evaluating and ranking existing biomarkers based on their sensitivity of prevalence.

Traditionally, researchers have tried to create strong biomarkers by combining together simpler ones through various hand-designed formulae^22,30,31^; This is the case for ABSI^18^ and RFM^19^. In contrast, here we propose to combine multiple raw biomarkers together through joint, multi-dimensional statistical models. We discover that 2D models (association of two biomarkers with one condition) yield higher discrimination and personalization than 1D models (association of one biomarker with one condition); and that they enable the separation of the negative effects of fat mass from the positive effects of muscle mass on people’s health risks.

Our results are validated on large datasets of participants (subsets of NHANES N ~ 100 K) and explained via new, compact, and intuitive visualizations.

## Methods

### Participants

All analyses in this study were conducted using the NHANES dataset^32^, collected by the Center for Disease Control and Prevention (CDC) between the years 1999 and 2020. The dataset comprises a total of more than 100,000 unique participants with data related to: demographics, body composition, fitness habits, eating habits and medical conditions. Our analysis focuses on the adult population only (ages between 20 and 110). The Supplementary Material available online presents a detailed accounting of the NHANES study design, participant selection, sample size and participants demographic characteristics.

### Health conditions

This study considers 6 common health conditions: hypertension, diabetes, high cholesterol, arthritis, coronary heart disease and cancer (general malignancy). Being positive to a condition is assessed via participants’ own answers to questions like: “Has a doctor ever told you that you have diabetes?”, as defined in the NHANES protocol (see Supplementary Material). The lack of an official diagnosis likely adds noise to our results, but aggregating statistics over a relatively large number of participants mitigates that issue.

### Body composition biomarkers

This study analyzes the predictive power of 23 biomarkers, amongst which: BMI, WHR, ABSI, PBF and RFM. The full list of biomarkers and their description is in the Supplementary Material. We have organized all biomarkers into three groups: global body composition biomarkers (e.g., BMI, PBF, total body weight), composition biomarkers based on regional measurements (e.g., percent trunk fat, waist circumference, WHR), and biomarkers that are less strongly associated with body composition (e.g., standing height and leg length).

### Statistical models for risk change prediction

**Figure 1:**
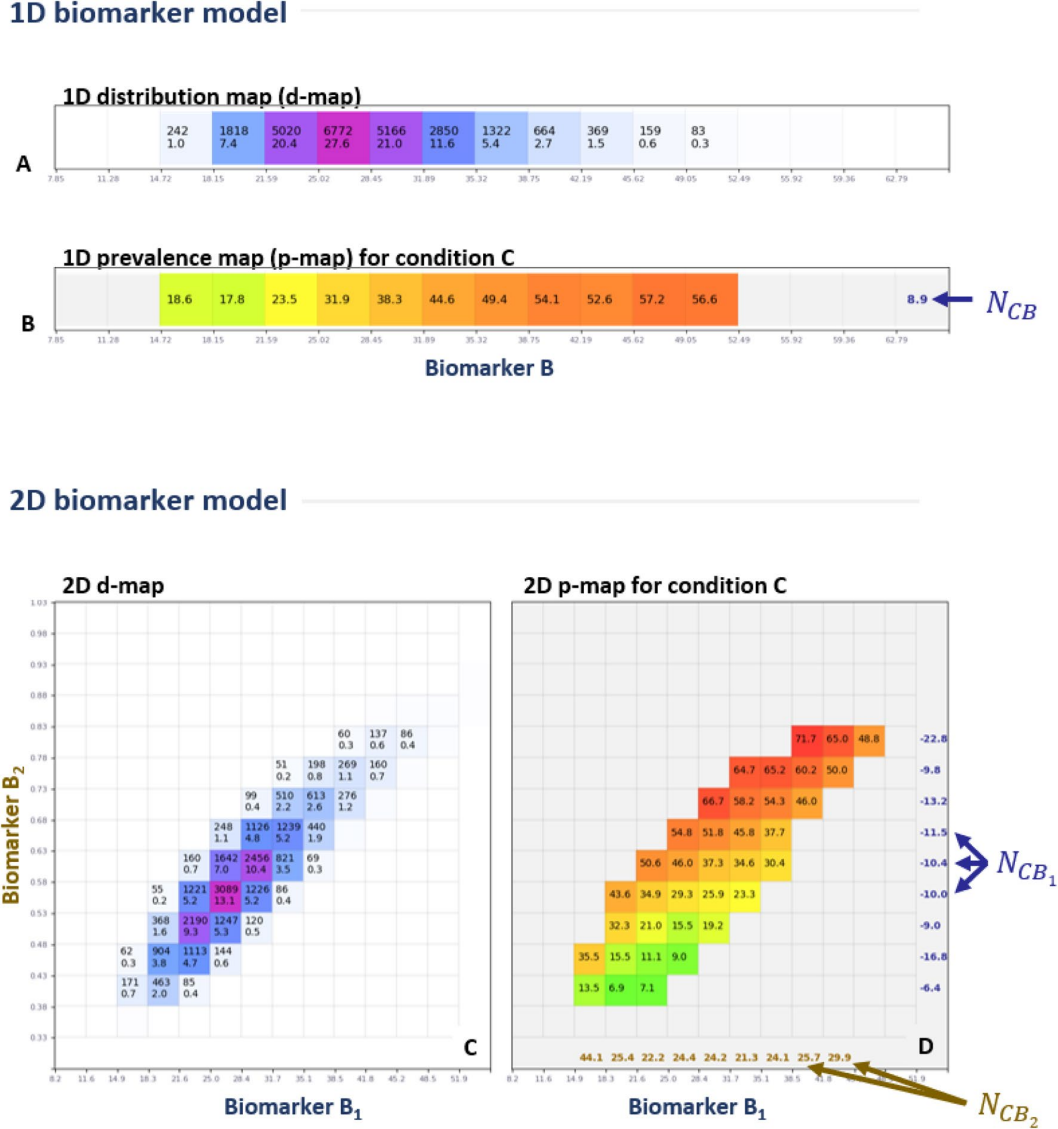
1D and 2D biomarker models. (**A**) A 1D d-map for a given population. (**B**) The corresponding 1D p-map for a condition C of interest. (**C**) A 2D d-map for a given population. (**D**) The corresponding 2D p-map. NORSE scores are indicated at the end of each row and column.

Here we present two different types of risk change prediction models. 1D models are those where we study the association between one medical condition and one input biomarker. In 2D models, we have one medical condition and two input biomarkers. Multi-dimensional models combine multiple biomarkers using a joint statistical model, rather than trying to compress their information into a single, scalar output. Examples of 1D and 2D biomarker models are illustrated in Fig. 1. Notice that in theory, it is possible to extend our models to a dimensionality higher than 2. However, the limited amount of data in NHANES and the so called “curse of dimensionality” would yield noisier results^33^.

### Distribution and prevalence maps

Our biomarker models are visualized via two types of visualizations: “population distribution maps” (aka *d-maps*) and “condition prevalence maps” (aka *p-maps*).

#### d-map

A distribution map reports the probability distribution of a population as a function of input biomarkers (Fig. 1A, C). Each cell in a d-map reports the total number of people with the biomarker $B$ within a given range (e.g.,$B \in$ [18.5, 25]), both in absolute terms (e.g., $n_{cell} = 5166$ participants) and as a percentage of the total population (e.g., $\frac{{n_{cell} }}{{n_{tot} }} = 21.0\%$). A d-map is visualized via a white-blue-purple colormap where white denotes 0% and purple denotes the maximum probability for that map.

#### p-map

A prevalence map reports the prevalence of a given medical condition $C$ as a function of a biomarker $B$ (Fig. 1B, D). We have $n_{cell}$ participants in a cell, out of which $n_{cond}$ are positive for the condition $C$. The cell reports the condition prevalence $P_{C} = \frac{{n_{cond} }}{{n_{cell} }}$ as a percentage. A p-map is visualized via a grey-green-yellow–red colormap, with green indicating low prevalence and vice-versa for red. In d-maps and p-maps cells with $n_{cell} < 40$ or $\frac{{n_{cell} }}{{n_{tot} }} < 0.2\%$ are left empty to reduce noise associated with small counts.

### Sensitivity of prevalence with respect to input biomarkers

In this study we explore associations between changes in biomarkers (the independent variable $B$), and the corresponding change in condition prevalence (the dependent variable $P$). For this, we use derivative-based sensitivity analysis^34^. Illustrative examples are presented in Fig. S2 of the Supplementary Material. For a given amount of change $\Delta B$ in the $X$ axis, the corresponding change $\Delta P_{C}$ in $Y$ depends on the slope of the curve at that point (it is a local analysis). Sensitivity is defined as the partial derivative $S_{CB} = \frac{{ \partial P_{c} }}{ \partial B}$ (in the continuous domain). The higher the $S_{CB}$ value, the larger the influence of the input biomarker onto the condition prevalence. More generally, ${\varvec{B}} \in {\text{R}}^{{\text{n}}}$ is an n-dimensional biomarker vector, and ${\varvec{S}}_{CB}$ is the associated gradient vector ${\varvec{S}}_{CB} = \left[ {\frac{{ \partial P_{c} }}{{ \partial B_{1} }},\frac{{ \partial P_{c} }}{{ \partial B_{2} }}, \ldots ,\frac{{ \partial P_{c} }}{{ \partial B_{n} }}} \right]$.

Derivatives and gradients capture only *local* sensitivity of functions with respect to independent variables. However, our experiments show a roughly linear relationship between disease prevalence and various biomarkers, thus justifying our approach (see examples in Fig. S3 of the Supplementary Material).

### Normalized sensitivity to predict risk changes

In general, different biomarkers have different measurement units and vary in their value ranges. For example, for the standing height biomarker, we typically have $B \in \left[ {140,{ }220} \right]\;{\text{cm}}$ for adults, while for BMI we have $B \in \left[ {10,{ }60} \right]\;{\text{kg/m}}^{2}$. To compare sensitivities of diverse biomarkers with one another we first need to map their values to a canonical range. We do so via a *normalized* sensitivity score (namely NORSE) which we define as follows. A biomarker $B$ measured in our population has mean $\upmu _{B}$ and standard deviation $\sigma_{B}$. Thus, its z-score^35^ is $X = \frac{{B -\upmu _{B} }}{{\sigma_{B} }}$. The z-score of a measurement represents its distance (in terms of number of standard deviations) from the mean. By the chain rule, the sensitivity with respect to the z-score $X$ (i.e., the NORSE measurement $N_{CB}$) is defined as $N_{CB} = \frac{{ \partial P_{c} }}{ \partial B}\frac{ \partial B}{{ \partial X}} = \sigma_{B} S_{CB}$. The NORSE score is a unit-less number and can now be used to compare the risk predictive power of diverse biomarkers with respect to one another.

#### Normalized sensitivity in maps

For consistency and to aid comparisons, the length of the sides of each cell in our visualization maps are set to $\frac{1}{2} \sigma_{B }$ (see Fig. 1). The blue and brown numbers on the side of a p-map are the NORSE scores computed for each row and column, respectively. Small NORSE values ($\left| {N_{CB} } \right| < 2$) are hidden to remove noise in the visualizations. Notice how in the example in Fig. 1D the $N_{{CB_{1} }}$ sensitivities (blue) are negative, while the $N_{{CB_{2} }}$ ones (brown) are positive. This important effect will be discussed in detail in the results section.

All methods were performed in accordance with the relevant guidelines and regulations.

### Meeting presentation

This work has not been published or presented elsewhere.

## Results

Our modeling approach yielded five main new findings: (1) waist and hip circumferences used either in a ratio or within a 2D joint model yield the strongest predictive power; (2) fat-derived biomarkers have a stronger predictive power than weight-related ones such as BMI and total body weight; (3) regional body fat biomarkers are more predictive of health risks than global fat measurements; (4) 2D biomarker models produce smaller and more homogeneous cohorts than 1D ones which, in turn, leads to higher sensitivity, discrimination and personalization of health risks; and (5) 2D biomarker models help us explain the “obesity paradox” as the effect of controlling separately for fat mass and lean mass. These observations are enumerated upon in the sections that follow.

### Predicting health risk changes from individual biomarkers

The NORSE scores for 23 biomarkers and 6 medical conditions for adult men and women are shown in Table 1. The last column reports NORSE scores averaged across conditions and genders. Such values are used to rank list all biomarkers.

**Table 1:** Ranking of 1D biometric models.

Biomarker ranking	Men								Women								Average NORSE across genders
	Biomarker	Hypertension	Arthritis	Diabetes	High cholesterol	Coron. heart dis	Cancer	Average NORSE	Biomarker	Hypertension	Arthritis	Diabetes	High cholesterol	Coron. heart dis	Cancer	Average NORSE	
**Regional composition biomarkers**																	
1	WHR	14.3	10.8	11.5	12.4	3.3	2.7	**9.2**	WHR	13.4	9.6	7.9	10.6	1.2	3.2	**7.7**	8.4
2	WThR	12.6	11.6	8.3	7.6	4.6	5.1	**8.3**	WThR	10.6	9.3	9.4	8.7	1.3	2.4	**7.0**	7.6
3	ABSI	10.6	9.2	6.1	8.6	3.6	5.7	**7.3**	ABSI	7.5	7.6	4.9	7.2	1.4	2.8	**5.2**	6.3
4	RFM	11.9	7.8	7.0	9.0	2.1	1.6	**6.6**	RFM	12.6	9.2	7.5	5.7	0.6	1.0	**6.1**	6.3
5	WeThR	11.6	7.2	6.2	7.0	2.5	1.2	**6.0**	WeThR	12.5	8.3	8.2	3.5	0.7	1.0	**5.7**	5.8
6	WHtR	9.8	6.9	6.7	6.9	1.5	1.0	**5.5**	WHtR	11.3	8.2	7.3	3.6	0.4	1.1	**5.3**	5.4
7	BAI	9.7	6.1	6.6	6.2	2.1	1.6	**5.4**	BAI	9.8	9.1	5.0	3.9	0.9	0.9	**4.9**	5.2
8	PTF	10.5	6.4	4.8	9.2	2.0	1.7	**5.8**	PTF	9.5	7.4	4.1	5.4	0.6	1.0	**4.7**	5.2
9	Waist circ	9.8	6.4	6.6	6.5	0.9	0.7	**5.2**	Waist circ	11.3	7.5	6.5	3.0	0.5	0.4	**4.9**	5.0
10	Hip circ	10.8	7.4	4.9	6.6	0.6	1.5	**5.3**	Hip circ	9.0	8.9	5.1	1.6	0.3	0.1	**4.2**	4.7
**Global composition biomarkers**																	
11	PBF	8.7	6.0	4.3	6.2	1.4	1.6	**4.7**	PBF	9.6	7.5	2.7	5.2	0.3	0.9	**4.4**	4.5
12	FTL	8.4	5.8	4.0	6.4	1.4	1.8	**4.6**	FTL	9.6	7.6	2.8	4.9	0.3	0.9	**4.4**	4.5
13	FMI	7.4	3.2	4.6	4.7	0.7	0.4	**3.5**	FMI	9.7	6.7	3.7	2.9	0.0	0.4	**3.9**	3.7
14	PI	8.7	4.2	5.1	5.0	0.8	− 0.1	**4.0**	PI	8.4	6.8	5.2	0.9	0.2	− 0.4	**3.5**	3.7
15	BMI	7.9	4.6	5.3	4.4	0.7	− 0.3	**3.8**	BMI	8.4	5.3	5.4	0.1	0.0	− 0.7	**3.1**	3.4
16	Weight	6.8	4.1	4.5	2.9	0.2	− 0.7	**3.0**	Weight	6.3	3.8	4.1	− 0.2	− 0.3	− 0.8	**2.2**	2.6
17	FFMI	5.6	1.5	3.2	3.8	− 0.1	− 1.5	**2.1**	FFMI	8.1	3.9	4.9	− 0.1	− 0.2	− 0.5	**2.7**	2.4
**Non compos. biomarkers**																	
18	Upper arm len	5.5	3.7	2.0	2.3	0.8	1.8	**2.7**	Upper arm len	5.4	3.8	2.1	0.3	0.1	0.6	**2.1**	2.4
19	Bicep circ	4.9	1.7	3.0	1.8	− 0.6	− 1.7	**1.5**	Bicep circ	7.6	3.8	4.4	1.8	− 0.1	− 1.2	**2.7**	2.1
20	Max calf circ	2.6	− 0.1	0.8	1.4	− 1.1	− 2.4	**0.2**	Max calf circ	0.9	− 0.7	− 1.0	− 3.2	− 0.7	− 2.0	− **1.1**	− 0.5
21	Thigh circ	1.8	− 1.8	− 0.7	0.0	− 1.9	− 2.7	− **0.9**	Thigh circ	3.8	− 0.4	− 0.3	− 2.7	− 0.7	− 1.4	− **0.3**	− 0.6
22	Height	− 0.9	− 0.3	− 1.5	− 2.0	− 0.7	0.0	− **0.9**	Height	− 4.2	− 3.2	− 2.4	− 4.2	− 0.6	− 0.1	− **2.5**	− 1.7
23	Leg length	− 5.2	− 4.1	− 4.8	− 5.2	− 1.9	− 1.7	− **3.8**	Leg length	− 7.3	− 6.4	− 6.3	− 6.7	− 1.1	− 1.3	− **4.9**	− 4.3
	Avg NORSE	7.6	4.7	4.3	4.9	1.0	0.8		Avg NORSE	7.6	5.4	4.0	2.3	0.2	0.4		

Normalized sensitivities (NORSE) values calculated for 23 different biomarkers (in rows) and 6 different conditions (in columns), for adult men and women.

Average NORSE values for each gender are in bold.

According to these results, WHR is the strongest health predictor, in the sense that normalized changes to WHR are associated with the largest changes in condition prevalence. The waist-to-thigh ratio is second, ABSI is third and RFM is fourth. BMI is in the middle of the table and total body weight lower still. Standing height and leg length have slightly negative NORSE scores, suggesting that tall people with long legs are statistically associated with lower health risks.

Interestingly, the top performing biomarkers are all *regional* ones; specifically, measurements associated with abdominal fat (e.g., WHR, ABSI, RFM, PTF). In the middle of the table we have *global* composition biomarkers (e.g. PBF, FMI, BMI); and at the bottom, biomarkers that do not correlate much with body composition (e.g., standing height, leg length). NORSE scores were able to cluster all biomarkers into these three groups automatically. Note that PBF is the strongest of the global adiposity biomarkers.

The bottom row in the table reports column-wise average NORSE scores. Their value indicates which health conditions are “easier” to predict from individual biomarkers. In our results, hypertension shows the largest average NORSE, and cancer the lowest.

#### Age stratification analysis

**Figure 2:**
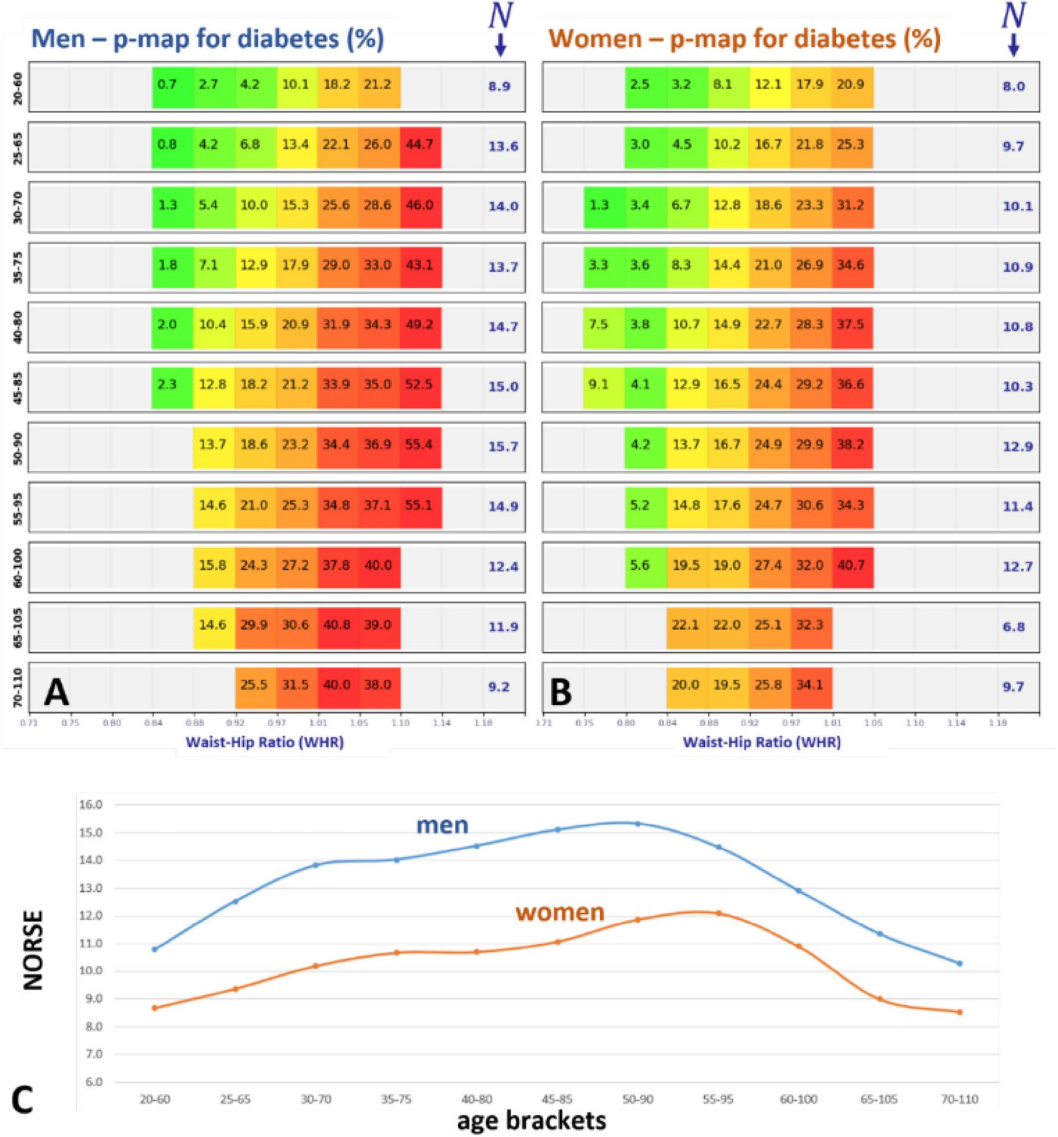
Age stratification of NORSE for C = diabetes in adult men (**A**) and women (**B**). (**C**) NORSE curve as a function of age.

As an example, the tables in Fig. 2 show diabetes prevalence with respect to WHR, for men and women and for different age brackets. As age increases diabetes prevalence increases, on average. The NORSE values follow a curve; they are low for young and elderly people, and they are higher in the middle. Very young people tend to have low diabetes risk even for high WHR values, and older people tend to have high prevalence, independent of WHR. People in the middle are those where changing WHR may have the greatest influence on their diabetes risk.

### Predicting health risk changes from joint, 2D biomarker models

**Figure 3:**
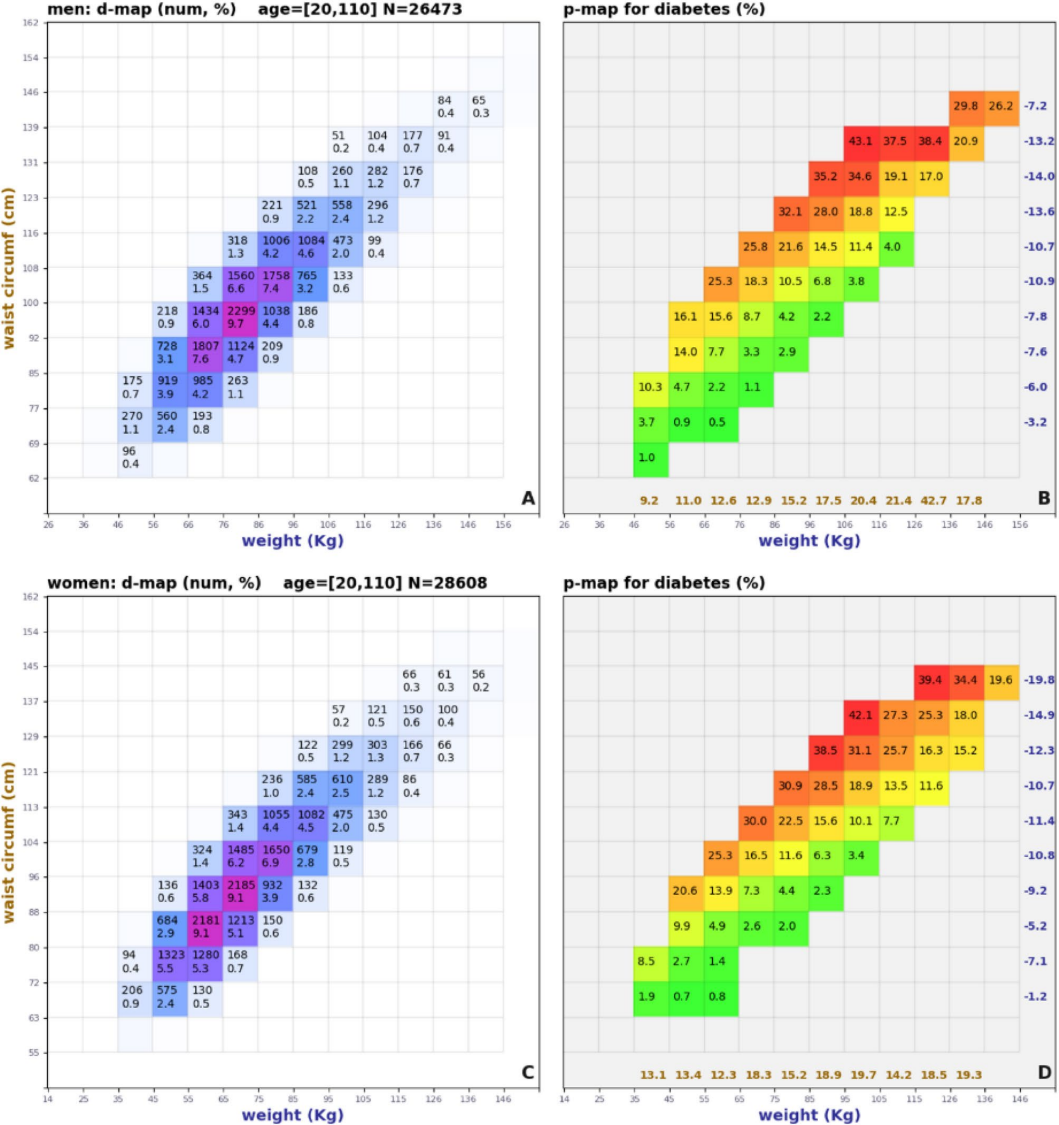
2D biometric models for X = weight, Y = waist circumference, C = diabetes for adult men (**A**, **B**) and women (**C**, **D**).

A 2D model associates two distinct biomarkers with the prevalence of a given condition. The example in Fig. 3 shows d-maps and p-maps for X = weight, Y = waist, C = diabetes for adult men and women. Notice that when fixing the weight coordinate (e.g., 66 < weight < 76 kg for men), diabetes prevalence increases considerably (from 0.5% to 25.3%) with increasing waist. Also, for a fixed waist (e.g., 100 cm < waist < 108 cm for men), prevalence *decreases* (from 25.3 to 3.8%) for increasing weight. This shows two things: (1) 2D biomarker models can discriminate different levels of risk better than using only one biomarker at a time, and (2) There are cases where increases in body weight correspond to improvements in health risks. Notice how all x-sensitivities (in blue) are negative, and all y-sensitivities (in gold) are positive.

#### Separating the effects of abdominal fat and lean mass

The effect of reduced health risks with (apparent) increased obesity goes under the name of the “obesity paradox”^36,37^. Here we explain the negative weight-risk correlation by separating the negative effects of fat from the positive effects of lean mass. In our 2D model, such separation happens naturally by controlling for waist circumference. All participants within the same row have a similar waist circumference. We hypothesize that for those people, residual weight increases are mostly due to increases in lean muscle tissue, which tends to be associated with better health^38–40^. With this interpretation the observed prevalence trends remain explained and there is no paradox.

#### Risk discrimination in 2D models

Two biomarkers can be combined together by e.g. taking a ratio (as for WHR) or through a joint 2D statistical model. In the former approach, some information is lost. In fact, imagine two people, one has waist = 81 cm, hip = 90 cm and the other has waist = 108 cm, hip = 120 cm. They have the same WHR = 0.9 but very different risk levels (see Fig. S5 in Supplementary Material). Generally, Multi-dimensional models yield higher risk discrimination than 1D ones, as shown next.

#### Ranking 2D biomarker models based on NORSE scores and NORSE separation

Our 23 biomarkers combine into 253 valid pairs. Each pair defines a 2D model, for which we measure its NORSE scores, across two genders and 6 health conditions. NORSE scores are calculated for both biomarkers (both along the x and along the y dimensions). For many models, one of those scores tends to be strongly negative (increasing biomarker correlates with reduced risks) and the other strongly positive (increasing biomarker correlates with increased risks). We hypothesize that their difference (namely *NORSE separation*) relates to the model’s ability to discriminate the negative effect of fat from the positive effect of lean mass.

Table 2 presents results for the 10 models with the largest NORSE separation. The right-most column reports average NORSE separations across conditions and genders. Those values are used to rank all biomarker pairs. Notice that for many 2D models, their average NORSE scores are higher than those of the 1D models (max avg NORSE is < 10 in Table 1, and > 19 in Table 2). In fact, keeping the input biomarkers separate (as opposed to fusing them together into a single output) allows us to subdivide the participants population into smaller and more homogeneous cohorts, for higher risk discrimination.

**Table 2:** Ranking of 2D biometric models.

Biomarker ranking	Men								Women								Average separation across genders
	Biomarker	Hypertension	Arthritis	Diabetes	High chol.	Cor heart dis	Cancer	Averages	Biomarker	Hypertension	Arthritis	Diabetes	High chol.	Cor heart dis	Cancer	Averages	
1	X hip	− 15.1	− 12.1	− 19.5	− 25.1	− 5.2	− 6.4	− **13.9**	X hip	− 15.1	− 9.8	− 10.6	− 16.1	− 1.3	− 1.1	− **9.0**	**28.4**
	Y waist	26.4	20.5	23.6	29.7	8.0	7.6	**19.3**	Y waist	25.3	20.5	14.0	19.5	3.5	4.0	**14.5**	
	NORSE sep	41.5	32.7	43.1	54.8	13.2	14.1	**33.2**	NORSE sep	40.4	30.2	24.7	35.6	4.8	5.1	**23.5**	
2	X thigh	− 19.6	− 16.7	− 13.7	− 10.7	− 8.3	− 9.1	− **13.0**	X thigh	− 16.2	− 14.3	− 9.7	− 17.6	− 3.2	− 6.7	− **11.3**	**25.6**
	Y BMI	21.0	17.9	11.3	16.5	7.0	5.3	**13.2**	Y BMI	22.7	20.2	16.0	18.0	3.4	1.6	**13.7**	
	NORSE sep	40.7	34.6	25.0	27.2	15.3	14.4	**26.2**	NORSE sep	38.9	34.5	25.7	35.6	6.6	8.4	**25.0**	
3	X weight	− 12.2	− 12	− 9.4	− 14	− 7	− 7.3	− **10.3**	X weight	− 12.2	− 12	− 10.3	− 13.1	− 3.2	− 5.2	− **9.3**	**24.8**
	Y waist	23.1	18.7	18.1	19.8	9.1	8.9	**16.3**	Y waist	22.7	18.3	16.3	17.3	2.7	5	**13.7**	
	NORSE sep	35.3	30.6	27.5	33.7	16.1	16.3	**26.6**	NORSE sep	34.9	30.3	26.6	30.4	5.9	10.1	**23.0**	
4	X weight	− 21.6	− 19.1	− 10.6	− 8.3	− 8.3	− 8.7	− **12.8**	X weight	− 13.7	− 15.2	− 13.4	− 11.1	− 1.4	− 4.7	− **9.9**	**24.4**
	Y thigh	20.4	19.1	10.5	13.3	7.0	9.1	**13.2**	Y thigh	19.9	19.1	13.4	16.9	2.2	5.7	**12.9**	
	NORSE sep	42.0	38.2	21.1	21.7	15.3	17.8	**26.0**	NORSE sep	33.7	34.4	26.8	28.0	3.7	10.3	**22.8**	
5	X PI	− 11.8	− 16.3	− 7	− 11.6	− 6.6	− 10.4	− **10.6**	X PI	− 7.3	− 5.2	− 9.3	− 13.2	− 2.8	− 4.6	− **7.1**	**23.3**
	Y WHtR	24.7	22.7	15.3	17.3	7.5	10.6	**16.4**	Y WHtR	18.1	15.9	15.1	14.8	3.3	7.6	**12.5**	
	NORSE sep	36.5	39.0	22.4	28.9	14.1	21.0	**27.0**	NORSE sep	25.3	21.1	24.4	28.1	6.1	12.2	**19.5**	
6	X BAI	− 11.6	− 13.5	− 6.9	− 15.7	− 3.2	− 6.5	− **9.6**	X BAI	− 6	− 1.8	− 9.8	− 10.8	− 1.6	− 0.5	− **5.1**	**22.7**
	Y WHtR	23.9	19.2	15.1	26.9	6.1	6.5	**16.3**	Y WHtR	25.9	19.5	13.1	19.2	2.4	6.8	**14.5**	
	NORSE sep	35.5	32.7	21.9	42.6	9.2	13.0	**25.8**	NORSE sep	31.8	21.3	22.9	30.0	4.0	7.3	**19.6**	
7	X PI	− 10.1	− 13.4	− 7.1	− 7.9	− 4.8	− 8.6	− **8.7**	X PI	− 7.1	− 6.9	− 4.7	− 8.0	− 2.3	− 6.4	− **5.9**	**22.6**
	Y RFM	26.7	25.4	19.5	18.6	8.8	10.1	**18.2**	Y RFM	18.1	17.3	15.4	16.0	2.2	6.1	**12.5**	
	NORSE sep	36.7	38.9	26.5	26.5	13.6	18.7	**26.8**	NORSE sep	25.3	24.1	20.1	24.0	4.5	12.5	**18.4**	
8	X hip	− 7.0	− 4.9	− 12.6	− 14.2	− 5.5	− 2.6	− **7.8**	X hip	− 11.8	− 12.7	− 10.6	− 15.7	− 1.3	− 1.5	− **8.9**	**22.5**
	Y WHtR	16.0	12.5	20.8	25.0	7.7	4.6	**14.4**	Y WHtR	23.9	17.8	13.7	18.7	3.4	4.9	**13.7**	
	NORSE sep	23.0	17.4	33.4	39.2	13.2	7.2	**22.2**	NORSE sep	35.7	30.4	24.3	34.4	4.8	6.5	**22.7**	
9	X BAI	− 10.1	− 12.7	− 8.7	− 14.8	− 3.8	− 6.2	− **9.4**	X BAI	− 7.9	− 3.1	− 6.5	− 13.0	− 1.2	− 3.7	− **5.9**	**22.3**
	Y RFM	22.5	16.9	18.0	21.5	5.5	6.9	**15.2**	Y RFM	25.2	17.0	16.6	18.2	2.7	4.5	**14.0**	
	NORSE sep	32.6	29.6	26.7	36.3	9.3	13.1	**24.6**	NORSE sep	33.1	20.1	23.1	31.2	3.9	8.2	**19.9**	
10	X BMI	− 11.7	− 15.4	− 9.4	− 7.8	− 7.1	− 8.4	− **10.0**	X BMI	− 7.5	− 8.7	− 6.8	− 12.1	− 2.9	− 6.2	− **7.4**	**22.2**
	Y WHtR	19.5	19.8	15.0	12.9	7.8	9.7	**14.1**	Y WHtR	18.4	17.6	14.4	17.3	2.9	7.7	**13.1**	
	NORSE sep	31.2	35.2	24.4	20.6	14.9	18.0	**24.1**	NORSE sep	25.9	26.3	21.3	29.4	5.8	13.9	**20.4**	

NORSE values calculated for 10 different biomarker pairs (in rows) and 6 health conditions (in columns), for adult men and women.

In average 2D models yield higher NORSE values than in 1D models.

Average values for each gender are in bold.

For both men and women, the largest NORSE separation is achieved by the hip–waist joint model. This confirms the power of using waist and hip circumferences for risk prediction (see Table 1).

#### The weight-waist 2D model

The NHANES dataset does not contain many measurements of hip circumferences (n = 2402 for men, n = 2523 for women valid measurements when intersected with C = hypertension). The pair weight-waist is amongst the best in terms of NORSE separation, but with one order of magnitude more measurements (n = 23,726 for men, n = 25,437 for women for C = hypertension). More data ensures lower measurement noise and more confident results. For that reason, our next example focuses on the weight-waist model.

**Figure 4:**
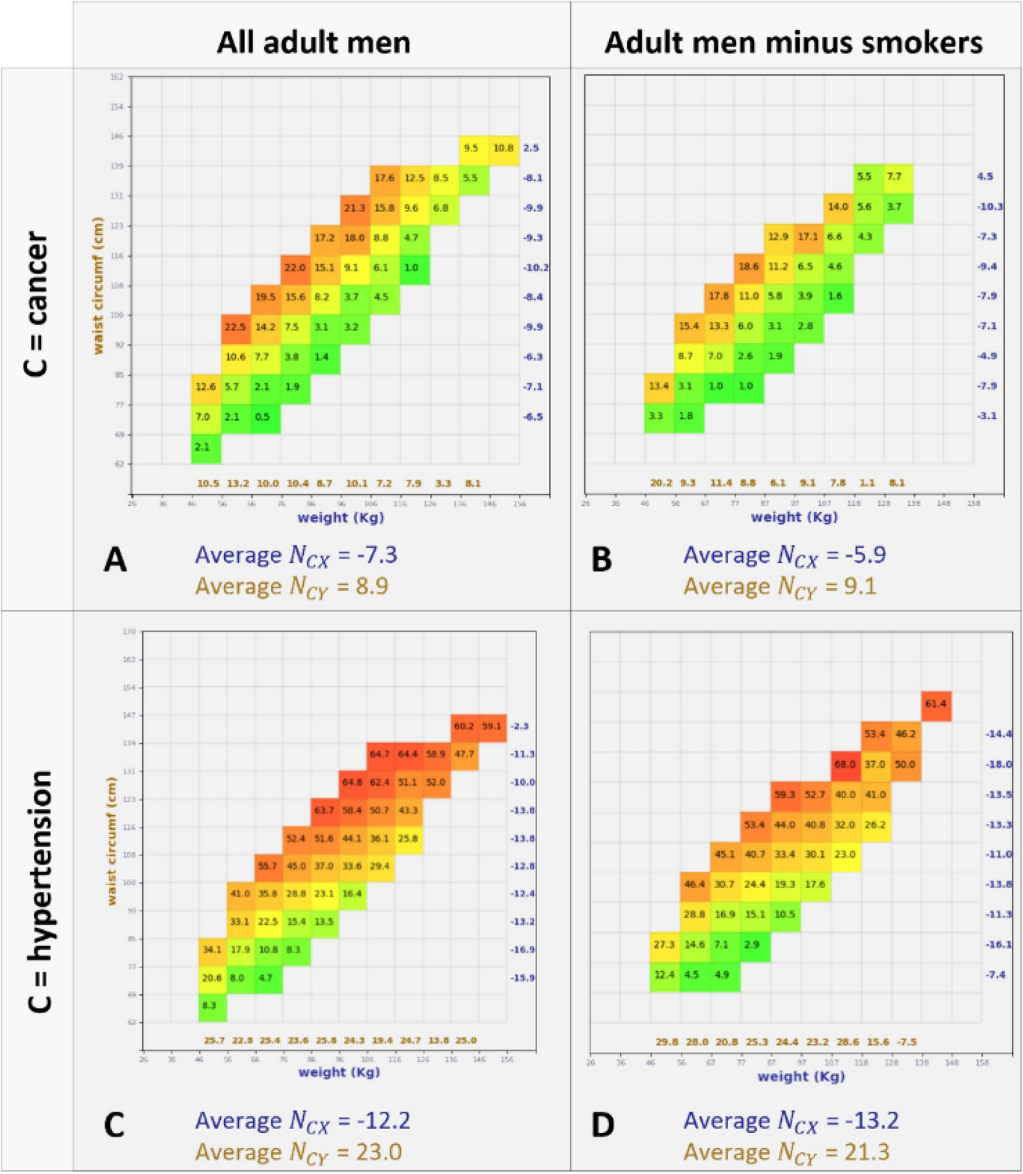
Prevalence maps and average NORSE scores for X = Weight, Y = Waist, C = cancer, for adult men. (**A**) including smokers. (**B**) excluding smokers. (**C**, **D**) Same as above but for hypertension.

Figure 4 shows p-maps for C = cancer (A, B) and C = hypertension (C, D) for adult men. In panel A, for a fixed weight the cancer prevalence increases with increasing waist circumference. When fixing the waist, the prevalence *decreases* with increasing weight. Panel B shows the same trends even after removing smokers from our analysis. Smokers here are detected through the SMQ020 NHANES code (“Smoked at least 100 cigarettes in life”). Similar results apply to hypertension (panel C, D), and same trends have been observed for the other four conditions, with or without smokers in the analysis. Age stratification results are presented in the Supplementary Material.

## Discussion

This study introduces a new way of assessing the strength of biomarkers as predictors of health risk changes. In contrast to AUC-ROC type techniques, here we estimate how much changes in input biomarkers affect changes in health risks. We achieve that through a new normalized sensitivity score.

The results in this paper show that when used in isolation, WHR is the biomarker with the strongest “effect” (in a sensitivity sense) on the risks of common health conditions. However, a high sensitivity also means that a small error in the input measurement is likely to have a large, detrimental effect on the accuracy of the output health risk.

For example, imagine that someone has waist = 85 cm and hip = 100 cm (thus WHR = 0.85), but those quantities are measured as waist = 87.5 cm, hip = 97.5 cm. Therefore, the WHR is erroneously measured as WHR = 0.9. A 2.5 cm error on the input biomarkers translates into a 0.05 error on the output WHR, which for adult men (Fig. 2A) translates into a large, 9% error on hypertension risk. These observations, exposed by the analyses reported herein, lead us to argue that to benefit from the increased sensitivity of our models, it is necessary to use state-of-the-art digital anthropometrics technology to increase input accuracy and thus the accuracy of risk predictions. Much literature discusses errors of measurements obtained using a measuring tape for example^41–43^. Recent progress in computer vision and photogrammetry offers accurate and inexpensive tools for measuring body composition and anthropometrics through optical scanners or even conventional smartphones^44–50^.

### Limitations

Limitations of the analysis presented here include: examination of cross-sectional data only, no longitudinal studies; establishing statistical associations rather than mechanistic understanding of cause and effect; lack of an official diagnosis for health conditions with reliance only on participants self-reported answers to a questionnaire; limited population size; treating diabetes as a single condition without distinction between type I and type II (by far the most common); and use of disease prevalence as a proxy for health risks.

## Conclusions

This study advances a new way of estimating the power of different body composition biomarkers when predicting health risk changes. Our results indicate that waist and hip circumferences, either used in a ratio or in a joint 2D model, hold the strongest predictive power. In general, regional body composition biomarkers produce the best results. We also show how joint biomarker models provide further resolution, prediction accuracy and the possibility to separate the negative effects of body fat from the positive effects of muscle mass. Our joint models help explain the “obesity paradox” via conventional statistical analysis.

We believe that our findings will lead to a better understanding of obesity, its causes and its effects on people’s health. Also, focusing on sensitivity measures may help individuals understand what behavior changes affect their health the most, and embrace healthier habits. Finally, combining our findings with emerging technology for body scanning and anthropometrics measurements promises to advance the way we assess obesity and associated health risks for everyone.

## Supplementary Information

Supplementary Information.

## Data Availability

The data used in this study can be downloaded from the Center for Disease Control and Prevention website at https://www.cdc.gov/nchs/nhanes/index.htm.
