# Supplementary material for: Normalized sensitivity of multi-dimensional body composition biomarkers for risk change prediction
Source: Sci Rep. 2022 Jul 20;12:12375. doi: 10.1038/s41598-022-16142-1 (PMC9300600; doi:10.1038/s41598-022-16142-1)
Supplement: Supplementary file 1 — Supplementary Information. [file 41598_2022_16142_MOESM1_ESM.docx]

**Normalized Sensitivity of Multi-Dimensional Body Composition Biomarkers for Risk Change Prediction**

**SUPPLEMENTARY MATERIAL**

A. Criminisi PhD ^1^, N. Sorek ^2^, S.B. Heymsfield, MD ^3^

^1^Amazon, Cambridge, UK; ^2^Amazon, Tel-Aviv, Israel, ^3^Amazon, Seattle, USA; Pennington Biomedical Research Center, Louisiana State University System, Baton Rouge, USA

We present here additional explanatory material in support of our main study.

**Data and Participant Characteristics**

The data used in this study comes from the National Health and Nutrition Examination Survey (NHANES) 1999-2018 collection^[[1]](#endnote-2)^. NHANES provides a representative sample of the non-institutionalized United States population. The sample includes four main race/ethnic groups: Non-Hispanic Black, Non-Hispanic White, Mexican American and Other Hispanic. Race and ethnicity were self-reported. The NHANES protocols were approved by the institutional review board of the National Center for Health Statistics, Centers for Disease Control and Prevention, and all participants provided written informed consent.

Additionally, in our study we remove from further analysis people of age below 20 or above 110, and pregnant women. For each health condition of interest, we further remove all data with invalid or missing answers to the corresponding questionnaire. The full consort diagram is presented in **Figure S1.** The table below presents general statistics of the male and female populations analyzed in this study.


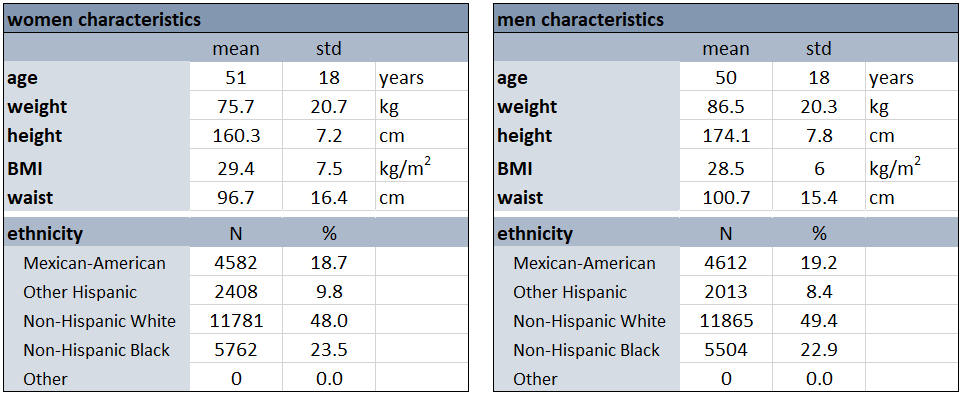


**Health Conditions Analyzed**

The table below presents the six health conditions analyzed here, their corresponding NHANES codes and the questions asked in the NHANES questionnaire.


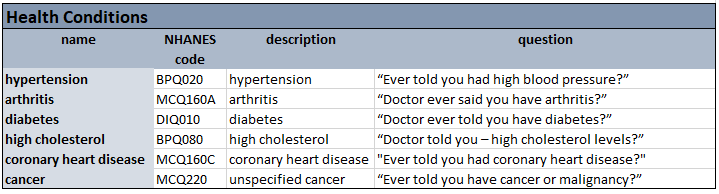


**Body Biomarkers Analyzed**

This study assesses the following 23 body biomarkers: Waist-to-Hip Ratio^[[2]](#endnote-3)^, Waist-to-Thigh Ratio, A Body Shape Index^[[3]](#endnote-4)^, Relative Fat Mass^[[4]](#endnote-5)^, Weight-to-Thigh Ratio, Waist-to-Height Ratio, Body Adiposity Index^[[5]](#endnote-6)^, Percentage Trunk Fat^[[6]](#endnote-7)^, Waist Circumference^[[7]](#endnote-8)^, Hip Circumference, Percentage Body Fat^[[8]](#endnote-9)^, Fat Mass to Lean Mass Ratio, Fat Mass Index^[[9]](#endnote-10)^, Ponderal Index^[[10]](#endnote-11)^, Body Mass Index^[[11]](#endnote-12)^, Body Weight, Fat Free Mass Index^[[12]](#endnote-13)^, Upper Arm Length, Upper Arm Circumference, Maximum Calf Circumference, Thigh Circumference, Standing Height, Leg Length. The OMNI online calculator^[[13]](#endnote-14)^ presents an exhaustive list of body biomarkers with a detailed description of each.

The NHANES codes used to compute our biomarkers are listed in the table below, together with a short description and measurement units. We have organized all biomarkers into three groups: global body composition biomarkers (e.g BMI, body weight, total percent body fat), regional composition biomarkers (e.g. percent trunk fat, waist-to-hip ratio, waist circumference), and biomarkers that are less strongly associated with body composition (e.g. standing height, arm length and leg length).


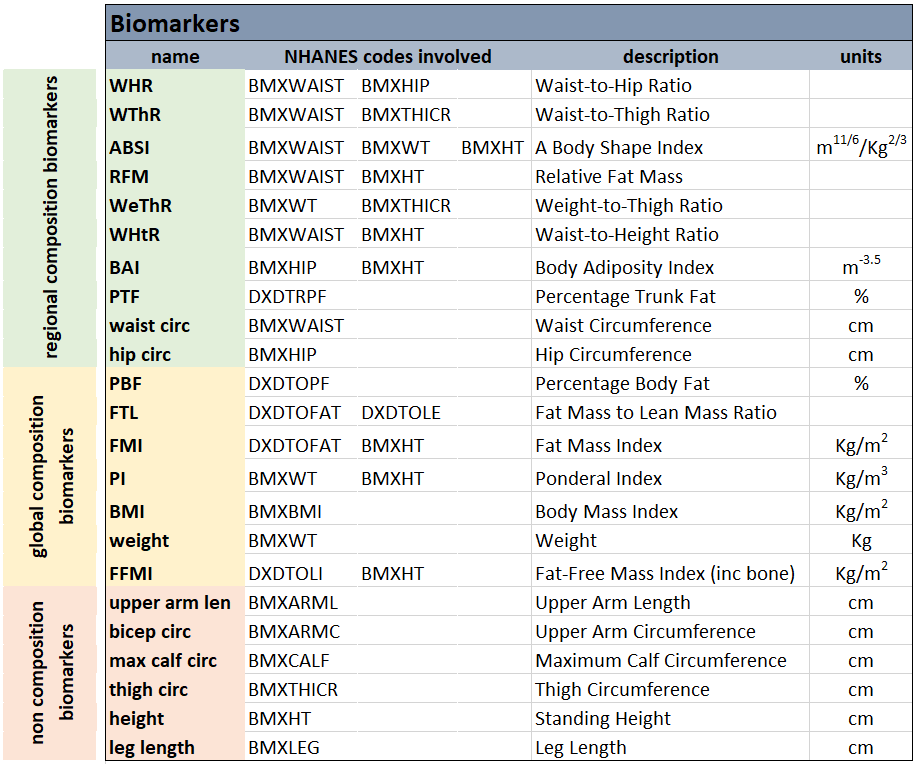


**Understanding Calculus-Based Sensitivity**

In the illustrative example in **Figure S2** we plot the prevalence of a condition in a given population as a function of two possible biomarkers, $B_{1}$ and $B_{2}$. The individual measurements are indicated with blue dots and the black line is fitted through them via Least Square Error^[[14]](#endnote-15)^. The sensitivity of the dependent variable $P$ with respect to the independent variable $B$ is defined as $S_{CB}=\frac{P_{c}}{B}$ . In discrete terms, this is approximated by $S_{CB}=\frac{\Delta P_{c}}{\Delta B}$. In this example, the sensitivity of (**A**) is the highest, in that a small change in $B_{1}$ yields the largest change in $P$.

**Normalized Sensitivity of Hypertension**

**Figure S3** shows examples of *normalized* sensitivity (NORSE) on real data. It shows d-maps and p-maps for adult women (age $\in$ [20,110]), and for the condition C=hypertension. The figure reports NORSE scores for: X=WHR (Fig. A, NORSE=13.4); X=BMI (Fig. B, NORSE=8.4); and X=weight (Fig. C, NORSE=6.3). Notice the roughly linear relationship between disease prevalence and biomarkers. According to these results, hypertension is most sensitive to changes in WHR (steepest line in the plot and largest NORSE score), and least sensitive to changes in overall weight (flattest line in the plot and lowest NORSE score).

**Age Stratification of Cancer Sensitivity in 2D Models**

**Figure S4** shows four p-maps for X=weight, Y=waist, C=cancer and gender=men. Each map corresponds to one of the following age brackets: [20,60], [35,75], [45,85] and [60,100]. We observe two facts: 1) The average NORSE score increases for increasing age. 2) For different age brackets the average weight NORSE remains negative and the average waist NORSE is positive. As observed earlier, the absolute value of NORSE scores is low for the younger and the older age brackets, and larger for ages in the middle (**Fig. E**); suggesting that middle aged people may benefit the most from reducing their waist size and/or building their muscles.

**Joint 2D Biomarker Models vs Composite 1D Models**

Multiple biomarkers can be combined together in different ways. For example, they could be plugged into more or less complex mathematical formulae to compute a single output; or they can be treated as distinct variables within a joint statistical model. The first approach yields loss of information, as explained next.

**Figure S5** shows a p-map for adult women, X=hip, Y=waist, C=hypertension. All the points along the dashed line have constant WHR=0.9; yet, they correspond to very different levels of risk (hypertension prevalence going from 15% to 50% along the line). This demonstrates inherent limitations of commonly used single biomarkers, and the added risk discrimination power of multi-dimensional biomarker models.


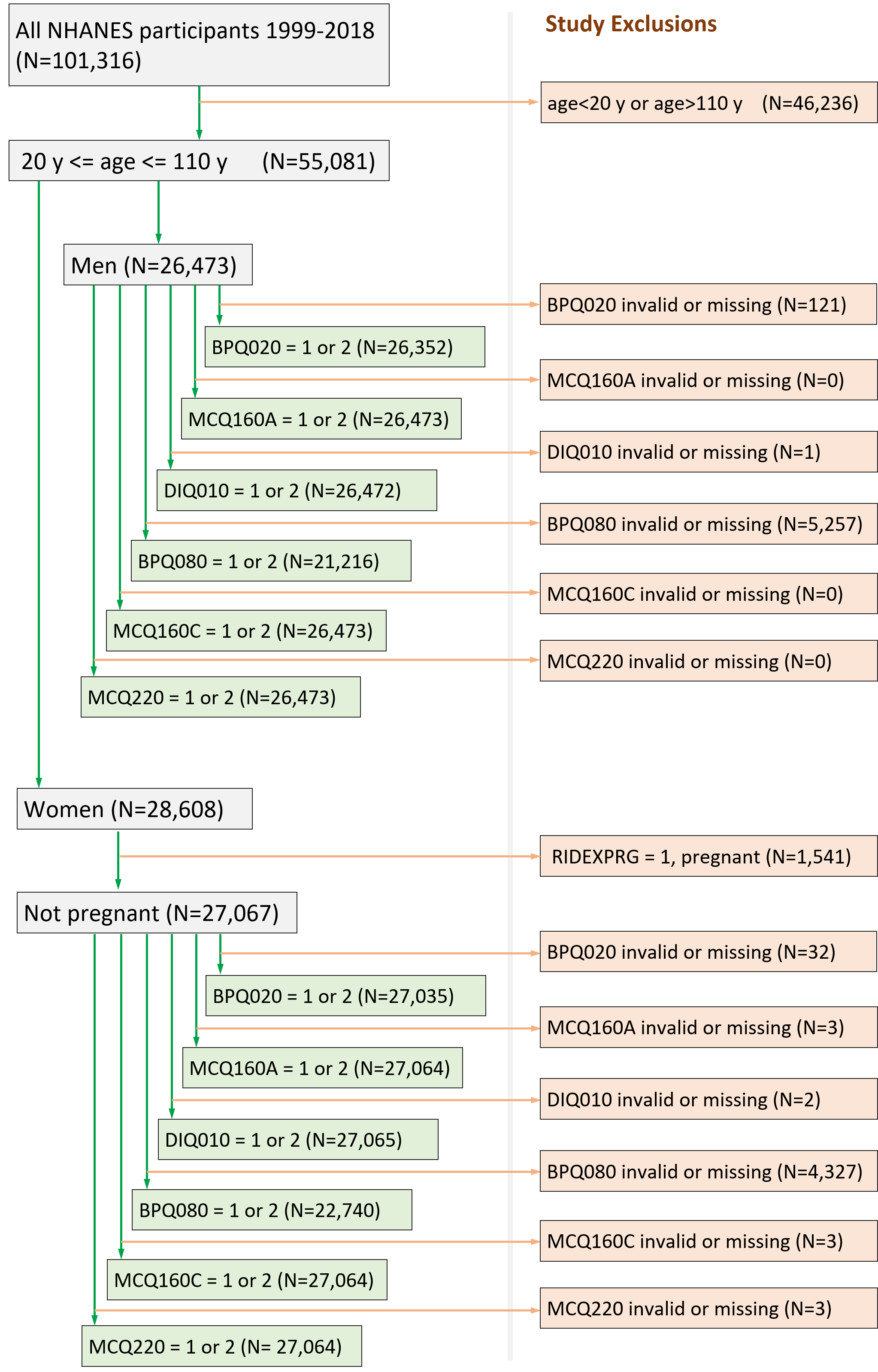


**Figure S1.** Consort diagram.


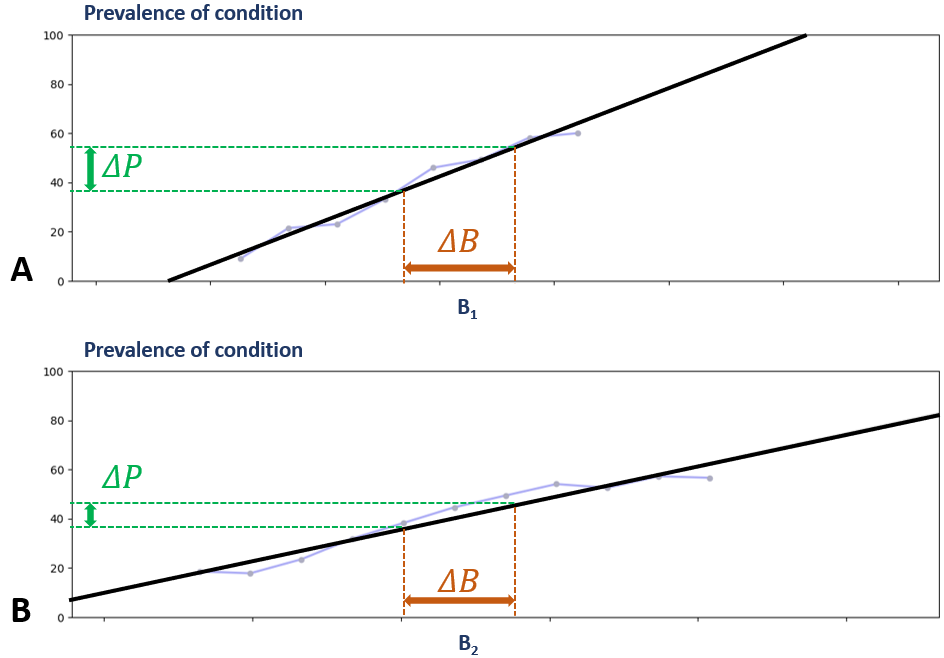


**Figure S2.** Illustrative prevalence curves for a given condition. The curve in (**A**) has higher sensitivity than the curve in (**B**) to changes in the independent variable B.

**
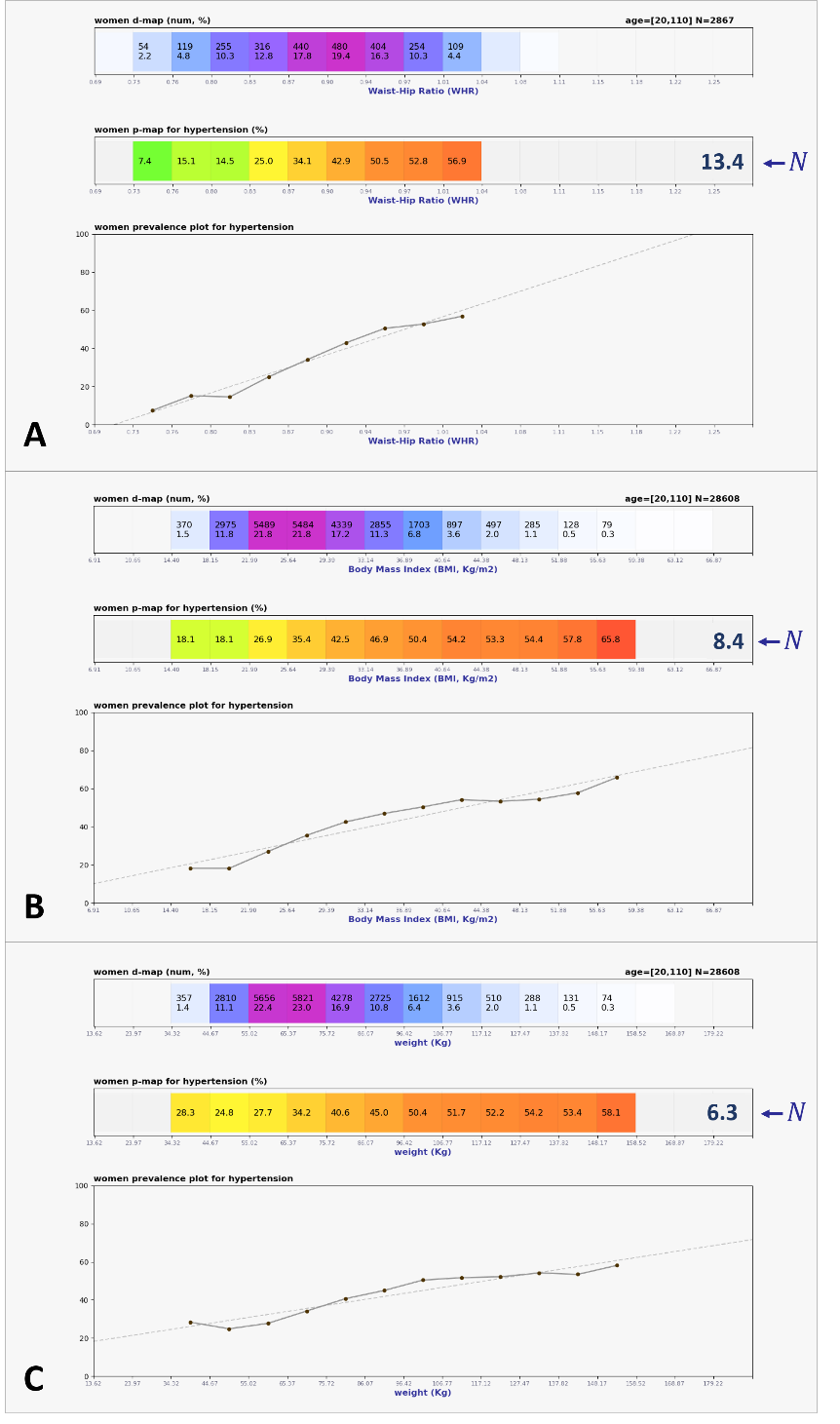
**

**Figure S3.** 1D d-map, p-map and prevalence curve for adult women, for C=hypertension and (**A**) X=WHR, (**B**) X=BMI, (**C**) X=Weight. Notice how hypertension prevalence is more sensitive to changes in WHR than to changes in other input biomarkers.

**
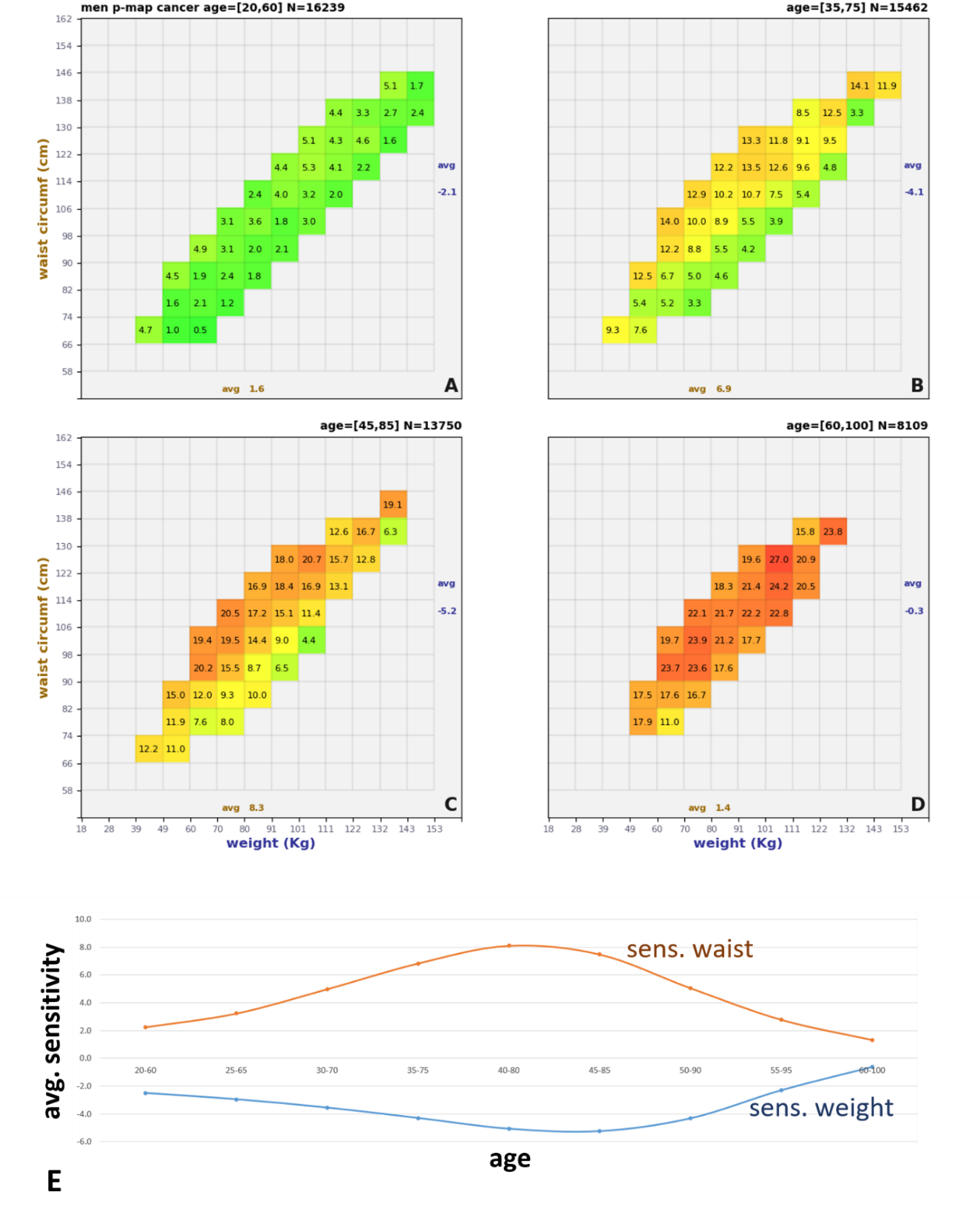
**

**Figure S4.** 2D prevalence maps and average NORSE scores for C=cancer, X=Weight, Y=Waist, for adult men in four different age brackets. (**E**) NORSE values are low both for younger and older individuals; but they increase (in absolute terms) for middle-aged people.


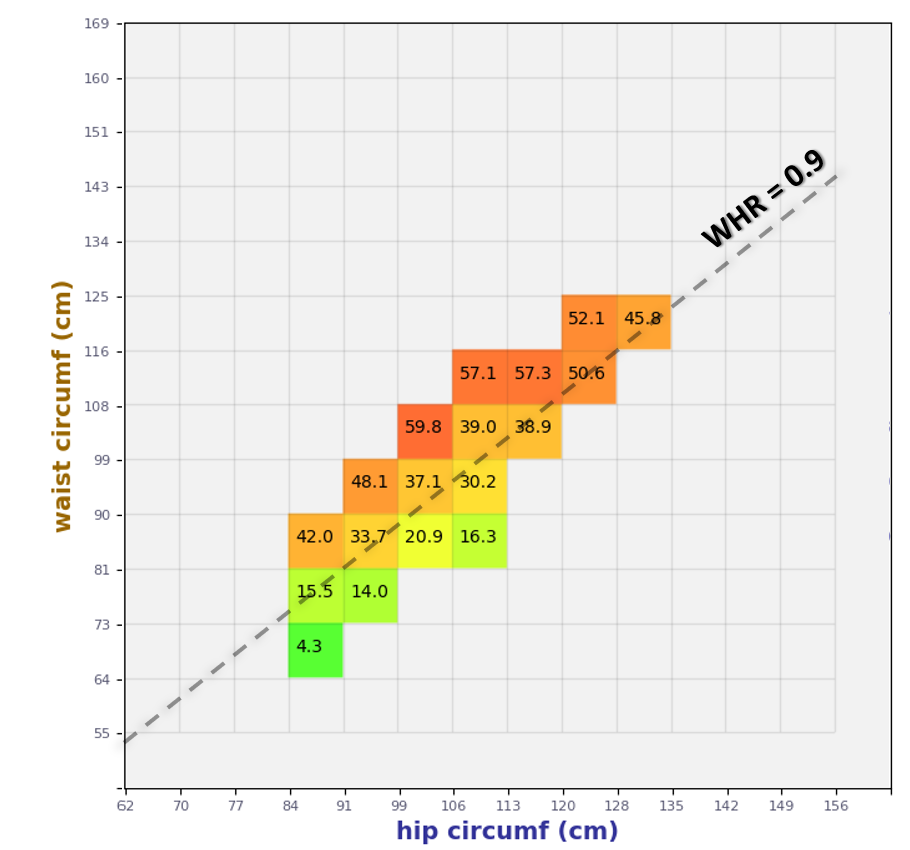


**Figure S5.** 2D p-map for adult women, X=hip, Y=waist, C=hypertension. All points along the dashed line have WHR=0.9. Despite the WHR being constant, points along the line go from a prevalence of 15.5% to more than 50%; thus demonstrating the added discrimination power of multi-dimensional biomarker models.

1. **References**

   Center for Disease Control and Prevention (CDC). National Health and Nutrition Examination Survey (NHANES) Homepage. Accessed Jan, 2022. <https://www.cdc.gov/nchs/nhanes/index.htm>. [↑](#endnote-ref-2)
2. World Health Organization (WHO). Waist circumference and waist-hip ratio: report of a WHO expert consultation. Geneva, 8-11 December 2008. [↑](#endnote-ref-3)
3. Christakoudi S, Tsilidis KK, Muller DC, Freisling H, Weiderpass E, Overvad K, Söderberg S, Häggström C, Pischon T, Dahm CC, Zhang J, Tjønneland A, Halkjær J, MacDonald C, Boutron-Ruault M-C, Mancini FR, Kühn T, Kaaks R, Schulze MB, Trichopoulou A, Karakatsani A, Peppa E, Masala G, Pala V, Panico S, Tumino R, Sacerdote C, Quirós JR, Agudo A, Sánchez M-J, Cirera L, Barricarte-Gurrea A, Amiano P, Memarian E, Sonestedt E, Bueno-de-Mesquita B, May AM, Khaw K-T, Wareham NJ, Tong TYN, Huybrechts I, Noh H, Aglago EK, Ellingjord-Dale M, Ward HA, Aune D, Riboli E. A Body Shape Index (ABSI) achieves better mortality risk stratification than alternative indices of abdominal obesity: results from a large European cohort. *Sci Rep* 2020. [↑](#endnote-ref-4)
4. Woolcott OO, Bergman RN. Relative Fat Mass as an estimator of whole-body fat percentage among children and adolescents: A cross-sectional study using NHANES. *Sci Rep* 2019. [↑](#endnote-ref-5)
5. Freedman DS, Thornton JC, Pi-Sunyer FX, Heymsfield SB, Wang J, Pierson RN Jr, Blanck HM, Gallagher D. The body adiposity index (hip circumference ÷ height(1.5)) is not a more accurate measure of adiposity than is BMI, waist circumference, or hip circumference. Obesity (Silver Spring). 2012. [↑](#endnote-ref-6)
6. He W, Zhang S, Song A, Yang M, Jiao J, Allison D, Heymsfield SB, Zhu S. Greater Abdominal Fat Accumulation Is Associated with Higher Metabolic Risk in Chinese than in White People: An Ethnicity Study. PloS one. 2013. [↑](#endnote-ref-7)
7. World Health Organization (WHO). Waist circumference and waist-hip ratio: report of a WHO expert consultation. Geneva, 8-11 December 2008. [↑](#endnote-ref-8)
8. Tomlinson DJ, Erskine RM, Morse CI, Onambélé GL. Body Fat Percentage, Body Mass Index, Fat Mass Index and the Ageing Bone: Their Singular and Combined Roles Linked to Physical Activity and Diet. *Nutrients*. 2019. [↑](#endnote-ref-9)
9. Schutz Y, Kyle UU, Pichard C. Fat-free mass index and fat mass index percentiles in Caucasians aged 18-98 y. Int J Obes Relat Metab Disord. 2002.  [↑](#endnote-ref-10)
10. Zaniqueli D, Oliosa PR, Neves FS, Pani VO, Martins CR, de Souza Peçanha MA, Rodrigues Barbosa MC, Rodrigues de Faria E, de Oliveira Alvim R, Mill JG. Ponderal index classifies obesity in children and adolescents more accurately than body mass index z-scores. *Pediatr Res* 2019.  [↑](#endnote-ref-11)
11. Centers for Disease Control and Prevention. Defining Adult Overweight and Obesity. Online. 2020. [↑](#endnote-ref-12)
12. Schutz Y, Kyle UU, Pichard C. Fat-free mass index and fat mass index percentiles in Caucasians aged 18-98 y. *Int J Obes Relat Metab Disord.* 2002. [↑](#endnote-ref-13)
13. https://www.omnicalculator.com/health [↑](#endnote-ref-14)
14. Lindstrom, D. [Schaum’s Easy Outline of Statistics](https://amzn.to/2O8E1Gy). McGraw-Hill Education. 2010 [↑](#endnote-ref-15)
